# Prognostic significance of the skeletal muscle index and systemic inflammatory index in patients with lymph node-positive breast cancer after radical mastectomy

**DOI:** 10.1186/s12885-022-09312-x

**Published:** 2022-03-03

**Authors:** Ru Tang, Jia-Peng Deng, Lei Zhang, Wen-Wen Zhang, Jia-Yuan Sun, Feng Chi, Jun Zhang, San-Gang Wu, Zhen-Yu He

**Affiliations:** 1 Department of Radiotherapy, Sun Yat-Sen University Cancer Center, State Key Laboratory of Oncology in South China, Collaborative Innovation Center for Cancer Medicine, 651 Dongfeng Road East, Guangzhou, 510060 People’s Republic of China; 2 Department of Radiation Oncology, National Cancer Center/Cancer Hospital, Chinese Academy of Medical Sciences (CAMS) and Peking Union Medical College (PUMC), Beijing, China; 3 Department of Radiation Oncology, Cancer Hospital, the First Affiliated Hospital of Xiamen University, Teaching Hospital of Fujian Medical University, Xiamen, 361003 China

**Keywords:** Skeletal muscle index (SMI), Systemic inflammation index (SII), Breast cancer, Overall survival

## Abstract

**Background:**

The role of skeletal muscle index (SMI) and systemic inflammation index (SII) for patients with lymph node-positive breast cancer remain controversial. This retrospective study aims to evaluate the individual and synergistic value of SMI and SII in outcomes prediction in this population.

**Methods:**

Lymph node-positive breast cancer patients who received mastectomy between January 2011 and February 2013 were included in this retrospective study. We used abdominal computed tomography (CT) to measure skeletal muscle mass at the third lumbar (L3) level. The optimal cut-off values of SMI and SII were determined through maximizing the Youden index on the receiver operating characteristic (ROC) curves. Kaplan–Meier method was used to assess the correlation between SMI, SII, and overall survival (OS). The prognostic value of SMI and SII were analyzed with the multivariable Cox proportional hazards model.

**Results:**

Of 97 patients included in our study (mean age: 46 [range: 27–73] years; median follow-up: 62.5 months), 71 had low SMI (sarcopenia), 59 had low SII, and 56 had low SMI + SII. Kaplan–Meier survival curves showed that both high SMI (*P* = 0.021, 5-year OS: 84.0% vs. 94.1%) and high SII (*P* = 0.043, 5-year OS: 81.0% vs. 97.3%) were associated with worse OS. Additionally, patients with either low SMI or low SII had significantly better OS (*P* = 0.0059, 5-year OS: 100.0% vs. 84.6%) than those with high SMI + SII. Multivariable analysis confirmed the predictive values of high SMI (*P* = 0.024, hazard ratio [HR]: 9.87) and high SII (*P* = 0.048, HR: 6.87) for poor OS. Moreover, high SMI + SII was significantly associated with poor survival (*P* = 0.016, HR: 16.36).

**Conclusions:**

In this retrospective analysis, both SMI and SII independently predicted the prognosis of patients with lymph node-positive breast cancer. SMI + SII might be a stronger prognostic factor than either alone based on our findings, but should be further verified in a larger study.

## Background

Skeletal muscle index (SMI) is an important parameter to measure the body composition. Low SMI indicates the presence of sarcopenia, a condition generally induced by multiple causes including lack of exercise, endocrine dysfunction, chronic diseases, systemic inflammation, and malnutrition [1]. A previous study found that sarcopenia correlates with the nutrition indicator such as prognostic nutritional index (PNI) [2]. In patients with cancer, sarcopenia is mostly observed during disease progression [3], and it is also associated with higher toxicities from chemotherapy, higher rates of treatment complications [4], and worse outcomes [5].

Chronic systemic inflammation is involved in tumor occurrence, development, and metastasis [6, 7]. Previous studies have shown that long-term inflammation may predispose to muscle loss and sarcopenia [8], which may aggravate systemic inflammation and lead to higher risk of mortality [9]. Therefore, biomarkers of systemic inflammation have unique values in predicting outcomes in patients with cancer.

Breast cancer is one of the most common cancers worldwide and the leading cause of malignant disease among women. With early detection and systemic therapies, patients with breast cancer can generally have a satisfactory outcome [10]. To achieve individualized treatment for breast cancer, it is vital to identify patients with potential adverse prognosis. Previous studies have noted an inverse relationship between SMI and chemotherapy toxicity in patients with either early-stage [11] or metastatic [12] breast cancer. Among patients with breast cancer who received postoperative adjuvant radiotherapy, SMI is also a promising prognostic indicator for predicting outcomes [13]. Indeed, low SMI indicates sarcopenia, which is associated with an increased risk of overall mortality in breast cancer survivors [5, 14–16]. Additionally, biomarkers of systemic inflammation such as systemic immune-inflammation index (SII), have shown their prognostic value in various malignant tumors [17–20] and are recommended as useful prognostic indicators for patients with breast cancer [21, 22]. However, the association of SMI and SII with survival in patients with lymph node-positive breast cancer remains to be determined.

In this study, we measured the skeletal muscle area at the level of the third lumbar vertebrae (L3) by computed tomography (CT) to calculate the value of SMI. We analyzed the prognostic values of SMI and SII in the survival of patients with lymph node-positive breast cancer. Our findings may offer guidance for developing individualized management and treatment programs for patients with lymph node-positive breast cancer.

## Methods

### Patient selection

In our retrospective study, we included 97 patients with lymph node-positive breast cancer who received surgical treatment between January 2011 to February 2013 at Sun Yat-sen University Cancer Center (SYSUCC). The follow-up data of each patient was collected through clinic visits or phone interviews. All data are complete at the time of analysis. The following inclusion criteria were applied: lymph-node metastasis; pathological diagnosis of stage II and III breast cancer; female sex; available abdominal CT images; and complete clinical data on age, body mass index (BMI), TNM stage, estrogen receptor (ER), progesterone receptor (PR), human epidermal growth factor receptor-2 (Her2), Ki67, complete blood count, and liver function tests. The exclusion criteria were as follows: distant metastasis; breast cancer recurrence; prior diagnosis of other cancers; other comorbidities that may affect survival such as cardiopathy and systemic immune disease; and prior usage of any immunosuppressants. The tumor staging was based on the International Union Against Cancer TNM classification system for breast cancer (7th edition) [23]. The classification of molecular subtypes was based on the guidelines established at the 13th St. Gallen International Breast Cancer Conference [18]. The Research Ethics Committee of SYSUCC approved this study. All patients provided written consent.

### The assessment of SMI and patient groupings

**Fig. 1:**
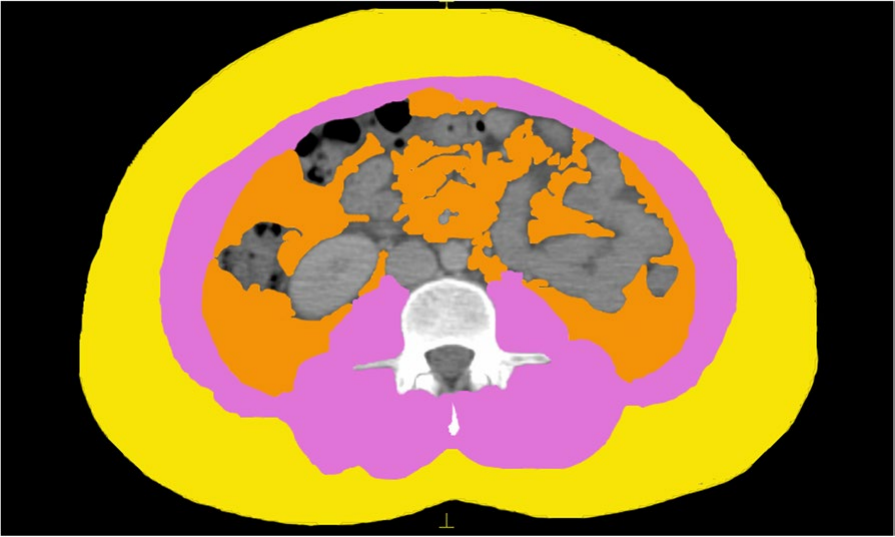
Body composition at the third lumbar vertebra level. Yellow: visceral adipose tissue (VAT); Pink: skeletal muscle mass (SMM); Orange: subcutaneous adipose tissue (SAT)

Abdominal CT images at the L3 level obtained from Monaco TPS version 5.1 (Elekta CMS, Maryland 72 Heights, MO, USA) before the surgical operation and chemotherapy was used to measure skeletal muscle area and fat tissue (Fig. 1). The measurement was based on their specific Hounsfield unit (HU) ranges. We calculated SMI as follows: SMI = skeletal muscle area (cm^2^)/height (m^2^).

Currently, there is no gold standard to define sarcopenia in cancer patients, and many studies generally seek to an approach that best separates patients with sarcopenia and those did not have sarcopenia with respect to the outcome of interest (i.e., mortality in this study). To this end, we defined patients with sarcopenia with the use of receiver operating characteristic curve (ROC) analysis, with the Youden index (sensitivity + specificity -1) being maximized to determine the optimal cut-off point of SMI. Because of the time-dependent nature of survival outcome data, we also used survival-ROC to determine the cut-off values and the results were similar. Patients with SMI value lower than the cut-point were categorized having sarcopenia. Similar categorization procedure was performed for SII, and prognostic nutritional index (PNI). In addition, we combined SMI with SII to obtain a new variable—SMI + SII. The low SMI + SII group includes patients with both low SMI and low SII values. The rest of the patients were included in the high SMI + SII group.

The PNI value was calculated using the following formula: PNI = serum albumin (g/L) + 5*peripheral blood lymphocyte count (*10^9^/L). The classification of BMI was based on the criteria specific for Chinese population: BMI < 18.5 kg/m^2^ (underweight), 18.5–23.9 kg/m^2^ (normal), 24–27.9 kg/m^2^ (overweight), and ≥ 28 kg/m^2^ (obese) [24, 25].

### Statistical analysis

We used Mann–Whitney U test to compare age, BMI, T stage, N stage, clinical stage, PNI, and SII between the high SMI and low SMI groups. The Pearson chi-squared test was used to compare ER, PR, Her2, Ki67 stage, and subtype. Overall survival (OS) was defined as the interval from the date of surgery to the date of death or latest follow-up. Survival curves were constructed with the Kaplan–Meier method, and the log-rank test was used for between-group comparisons. Univariable analysis was used to identify clinical variables associated with survival. Multivariable Cox proportional hazards regression models were used to adjust for clinically relevant variables from univariable analysis. All statistical analysis were performed in SPSS version 26.0 (IBM Corp., Armonk, NY) and R version 3.6.3 (R Foundation for Statistical Computing, Vienna, Austria). A two-sided *P* value < 0.05 was considered to indicate statistical significance.

## Results

### Clinical baseline data

The median age of the 97 included patients was 46 (range, 27–73) years. After a median follow-up of 62.5 months, a total of 8 deaths occurred. The baseline characteristics are shown in Table 1. The median BMI was 22.72 kg/m^2^ (range: 18.83–32.05 kg/m^2^). We measured the skeletal muscle area and calculated the SMI value (Fig. 1). The area under the ROC curve (AUC) determines the optimal cut-off points for SMI (44.91 cm^2^/m^2^, median: 41.62 cm^2^/m^2^, range: 21.1–55.53 cm^2^/m^2^); SII (610.79 × 10^9^/L, median: 445.22, range: 118.67–2434.89); and PNI (55.53 g/L, median: 52.2 g/L, range: 44–63 g/L) with an AUC value of 0.61, 0.56, and 0.65, respectively. Low SMI (sarcopenia) was observed in 71 (73.2%) patients and was associated with lower BMI (*P* < 0.001). A total of 59 (60.8%) and 56 (57.7%) patients were categorized into the low SII and low SMI + SII groups, respectively.

**Table 1:** Baseline characteristics of study patients

Variable	*N* (%)	Low SMI	High SMI	*P*
Age, years [mean: 45.69]				0.970
< 60	86	63	23	
≥ 60	11	8	3	
BMI				0.001
Normal (18.5–23.9)	66	48	18	
Overweight (24.0–27.9)	24	18	6	
Obese (≥ 28.0)	7	5	2	
T stage				0.087
1	24	15	9	
2	53	39	14	
3	10	8	2	
4	10	9	1	
N stage				0.772
1	30	22	8	
2	37	28	9	
3	30	21	9	
Clinical stage				0.987
II	26	19	7	
III	71	52	19	
ER				0.434
Negative	36	28	8	
Positive	61	43	18	
PR				0.715
Negative	44	33	11	
Positive	53	38	15	
Her2				0.423
Negative	57	40	17	
Positive	40	31	9	
Ki67				0.113
Negative	22	19	3	
Positive	75	52	23	
Subtype				0.764
Luminal A	11	6	5	
Luminal B	52	41	11	
Her2 +	21	15	6	
TNBC	13	9	4	
PNI				0.769
Low	68	49	19	
High	27	21	6	
Missing	2	1	1	
SII				0.548
Low	59	56	3	
High	38	15	23	

*SMI* skeletal muscle index, *ER* estrogen receptor, *PR* progesterone receptor, *Her2* human epidermal growth factor receptor-2, *TNBC* triple negative breast cancer, *PNI* prognostic nutritional index, *SII* systemic inflammation index

### Prognostic value and survival analysis

**Fig. 2:**
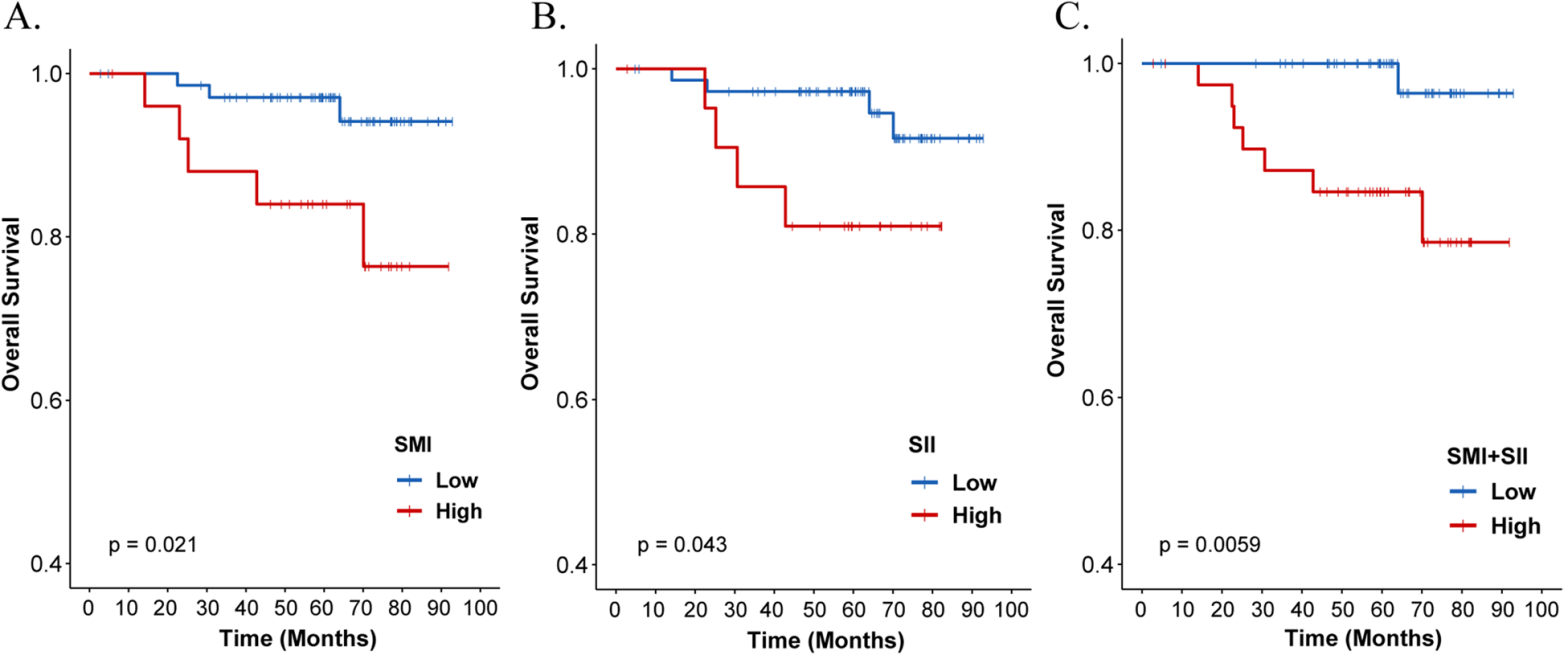
Kaplan–Meier curves indicating the overall survival (OS) rates of SMI (**a**), SII (**b**), and SMI + SII (**c**), respectively. SMI, skeletal muscle index; SII, systemic inflammation index

Kaplan–Meier curves demonstrated that patients with low SMI had more favorable OS than those with high SMI (*P* = 0.021, 5-year OS: 94.1% vs. 84.0%) (Fig. 2a). In terms of SII, patients with low SII had better OS than patients with high SII (*P* = 0.043, 5-year OS: 97.3% vs. 81.0%) (Fig. 2b). In addition, patients with both low SMI and low SII (low SMI + SII group) were associated with significantly better OS (*P* = 0.0059, 5-year OS: 100.0% vs. 84.6%) (Fig. 2c).

**Fig. 3:**
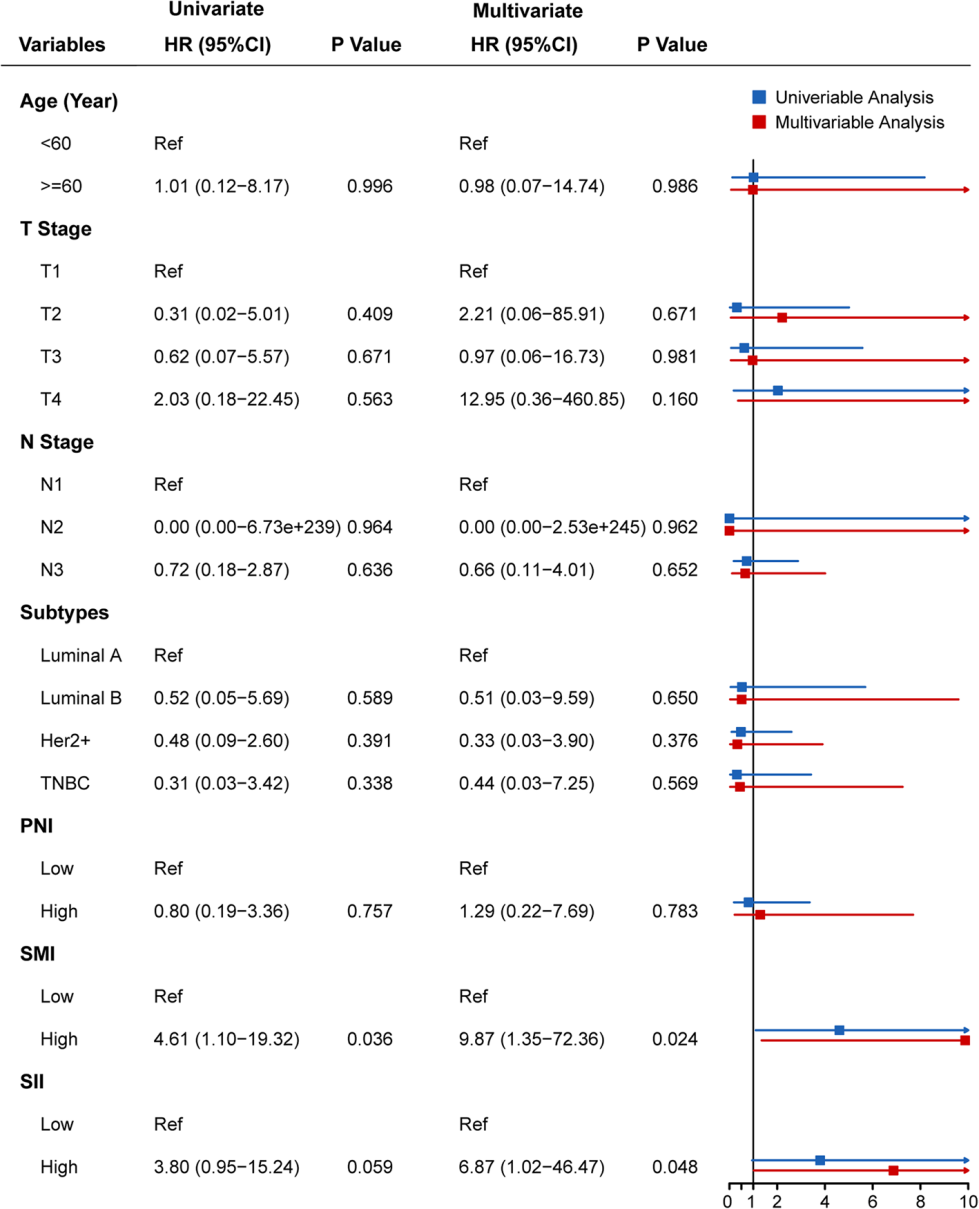
Cox proportional hazards models showing the correlation between clinical variables and overall survival (*n* = 97): Model A. PNI, prognostic nutritional index; SMI, skeletal muscle index; SII, systemic inflammation index

**Fig. 4:**
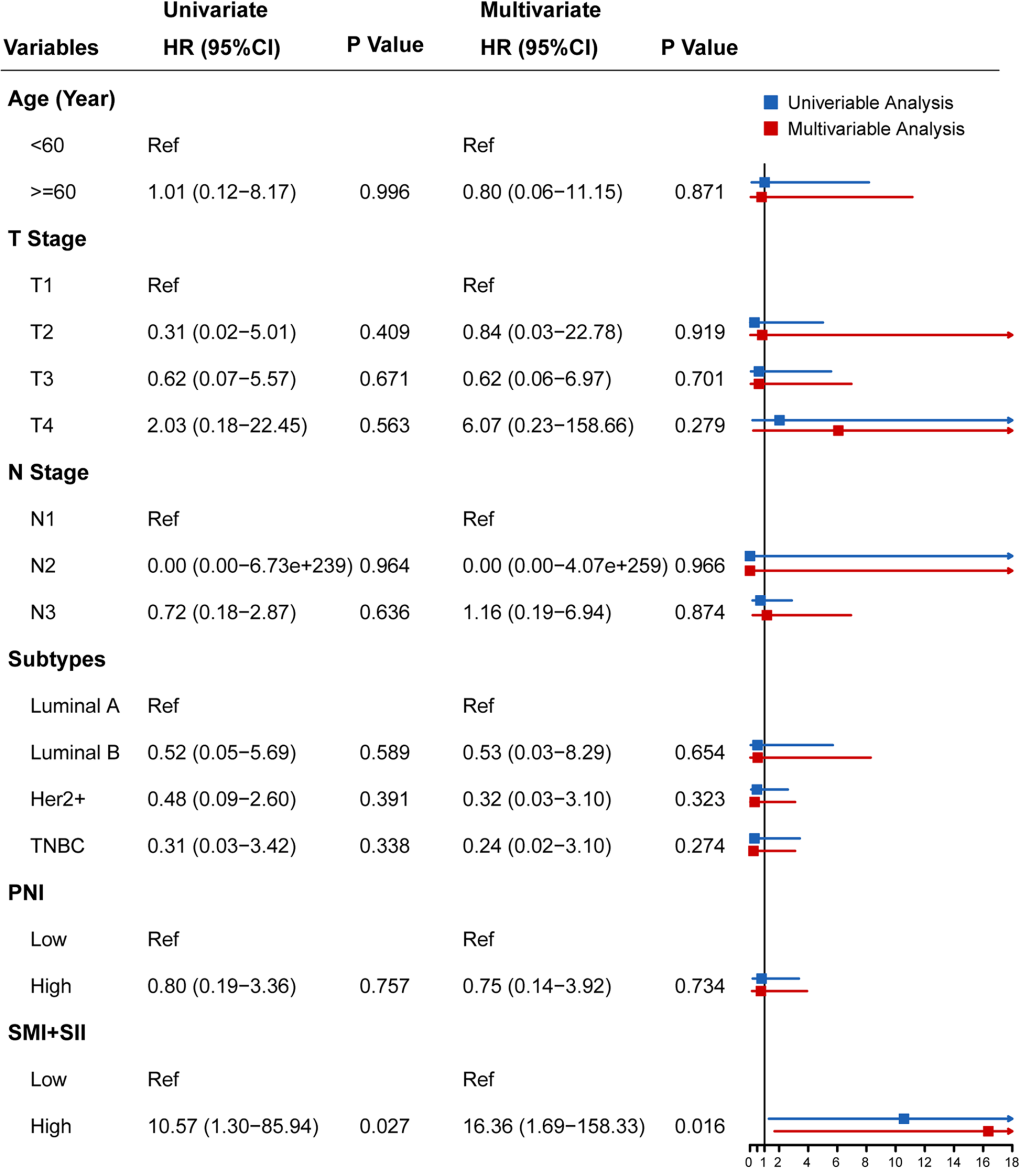
Cox proportional hazards models showing the correlation between clinical variables and overall survival (*n* = 97): Model B. PNI, prognostic nutritional index

Univariable Cox proportional hazards regression analysis (Figs. 3 and 4) showed that high SMI (*P* = 0.036; hazard ratio [HR]: 4.613, 95% confidence interval [CI], 1.101–19.322) and high SMI + SII (*P* = 0.027, HR: 10.571, 95% CI, 1.300–85.936) were significantly associated with worse survival. In the multivariable Cox proportional hazards regression models adjusted for clinical variables, high SMI (*P* = 0.024), high SII (*P* = 0.048), and high SMI + SII (*P* = 0.016) were independently associated with worse survival. Additionally, the high SMI + SII groups (HR [95% CI]: 16.361 [1.691–158.325]) had significantly higher risk of mortality than the high SMI (HR [95% CI]: 9.867 [1.346–72.355]) or high SII (HR [95% CI]: 6.870 [1.015–46.472]) groups.

## Discussion

In this study, we examined the SMI and SII prognostic values in a retrospective cohort of patients with lymph node-positive breast cancer without distant metastasis. The results indicate that lower SMI and SII values were both independently associated with more favorable OS in these patients, and the combination of SMI and SII might better predict the survival outcomes.

Our study cohort had several notable factors that may contribute to fewer deaths. First, most patients had a pathological diagnosis of certain molecular subtypes (type luminal A and B) that are shown to be associated with satisfactory prognosis in previous studies [26]. Second, the patients were relatively young, and all of them had received standardized treatment modalities in our hospital. Nonetheless, SMI and SII still emerged as an important prognostic factor in both univariable and multivariable analysis, indicating their unique value for this patient population.

Our finding that low SMI was probably associated with better survival was consistent with a previous report [27], but appeared different from other studies. Caan et al. [5] and Deluche et al. [28] reported that sarcopenia was associated with a higher mortality among patients with nonmetastatic breast cancer. For those with metastatic breast cancer, Franzoi et al. [29] observed that sarcopenia is an indicator for poor prognosis. Both patients with early and advanced stage breast cancer struggle with serious chemotherapy-related toxicities. We speculated that the association of SMI with cancer survival may differ among patients of different age groups who may suffer from different chemotherapy-related toxicities resulted from diverse skeletal muscle index. In our study, the median age of breast cancer patients was 45 years and 75% were aged < 55 years, which was relatively young compared with previous studies [5, 28, 29]. Of note, the onset of menopause in women is generally associated with decreased skeletal muscle mass and increased adipose tissue [28]. Older patients generally have more adipose tissue than younger patients, which can be associated with more toxicity from chemotherapies [5]. For older patients receiving chemotherapy, a higher SMI value indicates better nutritional status and is predictive of better survival. However, for younger patients, a higher SMI value may be associated with a higher level of substance exchange within capillaries in the muscle tissue [4, 11], which could predispose to the development of chemotherapy-induced toxicities. In this regard, the increased risk of toxicities from chemotherapy may outweigh the benefits associated with a high SMI. Therefore, low SMI could be beneficial in younger patients and confer better treatment response and tolerance of chemotherapy instead of higher toxicities.

Moreover, the European Working Group on sarcopenia recommends to evaluate the presence of both low muscle mass and low muscle function (strength or performance) to determine sarcopenia [30]. Versteeg et al. [31] demonstrated that higher muscle strength could be beneficial for the survival of old patients with advanced cancer. Therefore, clinicians should not only focus on the value of SMI, but also incorporate patients’ nutritional status, muscle strength, extent of weight loss, and the daily lifestyle into the comprehensive assessment of prognosis.

Inflammation is closely connected with cancer, and previous studies have explored the prognostic value of a combination of SMI and common inflammatory markers. Demirelli et al. [32] showed that several inflammatory markers are independent prognostic factors in patients with metastatic gastric cancer. Cho et al. [33] found that sarcopenia accompanied by higher neutrophil/lymphocyte ratio is associated with inferior OS and progression-free survival in patients with head and neck cancer. The SII is a systemic inflammatory marker that takes into account the role of lymphocytes, neutrophils, and platelets. Lymphocytes plays a significant role in the inhibition of tumor proliferation and migration [34]. Lymphocytes can not only prevent cytotoxic cell death [35], but also secrete IFN-γ and TNF-α to control tumor growth [36]. Lack of lymphocytes may lead to insufficient immunoreaction, and thus promote disease progression [37]. In contrast, neutrophils can secrete a series of inflammatory mediators [38], which could positively affect tumor growth and metastasis. Neutrophils can also impair the cytolytic ability of activated T cells and NK cells [39]. In addition, platelets activated by tumor-related inflammation accelerate tumor cell migration and protect tumor cells from immune attack [40]. In essence, the development and progression of tumors are dependent on the balance between tumor-promoting factors (neutrophils and platelets) and tumor-inhibiting factors (lymphocytes). The SII integrates these relative factors and is considered a better inflammatory marker than other inflammatory prognostic factors that reflect this balance, such as neutrophil-to-lymphocyte ratio (NLR) or monocyte-to-lymphocyte ratio (MLR). In this study, we found that the OS of patients with low SMI and SII were significantly better than those with both high SMI and high SII. Therefore, our finding adds to the growing body of evidence that shows the important value of combining SMI with SII to predict prognosis in patients with solid tumors.

The improved predictive value of SMI and systemic inflammatory markers may be based on the mutual effects between skeletal muscle and inflammation. Skeletal muscle can secrete cytokines such as IL-6, IL-8, and IL-15 in an autocrine, paracrine, or endocrine manner, which promotes systemic inflammation and subsequently tumor growth [41]. Meanwhile, during chronic inflammation, the elevated production of reactive oxygen species (ROS) or reactive nitrogen species (RNS) can damage proteins through carbonylation that can lead to irreversible protein modification [7]. In fact, the primary cause for skeletal muscle loss in breast cancer patients receiving chemotherapies is tumor-related cytokines and chronic inflammation rather than the chemotherapy itself [11]. In the present study, patients with a higher SII value may have had an increased level of proinflammatory cytokines, which promotes the development of chronic inflammation and worse survival.

This study has some limitations. Firstly, the retrospective nature of this analysis indicates that certain confounding factors may not have been accounted for owing to incomplete clinical information. Some important demographic factors such as socioeconomic status may affect patient survival. Secondly, the relatively small sample size and fewer deaths could have prevented us from identifying significant relationships for TNM stage and pathological and molecular subtype with patient prognosis. Finally, our study sample consists of a relatively young Chinese population and thus, our conclusions may not be directly applicable to patients with different demographics.

## Conclusions

In summary, this retrospective analysis suggested that high SMI and high SII could be associated with poor OS in a relatively young group of patients with lymph node-positive breast cancer without distant metastasis. In clinical practice, a combination of SMI and SII may provide better prognostic values in predicting the prognosis for these patients to some extent. This warrants further confirmation in studies with a larger patient cohort in the future.

## Data Availability

According to our institution’s regulation, the original data will not be shared.

## Abbreviations

SMI: Skeletal muscle index
SII: Systemic inflammation index
CT: Computed tomography
L3: The third lumbar
ROC: The receiver operating characteristic
OS: Overall survival
PNI: Prognostic nutritional index
BMI: Body mass index
ER: Estrogen receptor
PR: Progesterone receptor
Her2: Human epidermal growth factor receptor-2
TNBC: Triple negative breast cancer
HU: Hounsfield unit
AUC: The area under the ROC curve
HR: Hazard ratio
CI: Confidence interval
NLR: Neutrophil-to-lymphocyte ratio
MLR: Monocyte-to-lymphocyte ratio
ROS: Reactive oxygen species
RNS: Reactive nitrogen species
